# SEL1L-HRD1 ER-associated degradation regulates leptin receptor maturation and signaling in POMC neurons in diet-induced obesity

**DOI:** 10.21203/rs.3.rs-3768472/v1

**Published:** 2024-01-12

**Authors:** Hancheng Mao, Geun Hyang Kim, Ling Qi

**Affiliations:** 1 Department of Molecular & Integrative Physiology, University of Michigan Medical School, Ann Arbor, MI 48105, USA; 2 Department of Molecular Physiology and Biological Physics, University of Virginia, School of Medicine, Charlottesville, VA 22903, USA; 3 Present address: Regeneron Pharmaceuticals, Inc., 777 Old Saw Mill River Road, Tarrytown, New York 10591, USA

**Keywords:** SEL1L-HRD1 ERAD, POMC, diet-induced obesity, leptin signaling, leptin receptor, parabiosis

## Abstract

Endoplasmic reticulum (ER) homeostasis in the hypothalamus has been implicated in the pathogenesis of certain patho-physiological conditions such as diet-induced obesity (DIO) and type 2 diabetes; however, the significance of ER quality control mechanism(s) and its underlying mechanism remain largely unclear and highly controversial in some cases. Moreover, how the biogenesis of nascent leptin receptor in the ER is regulated remains largely unexplored. Here we report that the SEL1L-HRD1 protein complex of the highly conserved ER-associated protein degradation (ERAD) machinery in POMC neurons is indispensable for leptin signaling in diet-induced obesity. SEL1L-HRD1 ERAD is constitutively expressed in hypothalamic POMC neurons. Loss of SEL1L in POMC neurons attenuates leptin signaling and predisposes mice to HFD-associated pathologies including leptin resistance. Mechanistically, newly synthesized leptin receptors, both wildtype and disease-associated human mutant Cys604Ser (Cys602Ser in mice), are misfolding prone and bona fide substrates of SEL1L-HRD1 ERAD. Indeed, defects in SEL1L-HRD1 ERAD markedly impair the maturation of these receptors and causes their ER retention. This study not only uncovers a new role of SEL1L-HRD1 ERAD in the pathogenesis of diet-induced obesity and central leptin resistance, but a new regulatory mechanism for leptin signaling.

## Summary

Here we report that POMC-specific SEL1L-HRD1 ER-associated degradation is indispensable for leptin signaling in diet-induced obesity by controlling the turnover and maturation of nascent leptin receptor in the ER.

## INTRODUCTION

Hypothalamic neurons play important roles in the adaptation and mal-adaptation to pathophysiological conditions such as diet-induced obesity (DIO) and type-2 diabetes ^1–7^. Homeostasis in the endoplasmic reticulum (ER) regulates many physiological processes such as systemic inflammation, inter-organellar crosstalk and mitochondrial dynamics ^8–14^. It has been proposed that hypothalamic ER stress or unfolded protein response (UPR) may play a causal role in inflammation and leptin resistance in DIO and type-2 diabetes ^15–21^. However, others have reported a protective role of UPR in similar experimental settings ^22,23^. Hence, the significance of ER quality control pathways and its underlying mechanisms remain controversial.

In addition to UPR that respond to misfolded proteins in the ER, ER-associated protein degradation (ERAD) is a constitutively active and highly conserved process responsible for recruiting unfolded or misfolded proteins in ER for cytosolic proteasomal degradation ^24–31^. Among over a dozen of putative ERAD complexes, the SEL1L-HRD1 protein complex represents the most evolutionarily conserved ERAD branch where SEL1L/Hrd3p is an obligatory cofactor for the E3 ligase HRD1 ^28–30,32–34^. Recent studies using cell type-specific SEL1L or HRD1 knockout mouse models have revealed the patho-physiological importance of SEL1L-HRD1 ERAD in a substrate-specific manner ^35–42^. Particularly relevant to this study, SEL1L-HRD1 ERAD has been reported as indispensable for AVP and POMC neurons to control water balance and food intake via the maturation of prohormones, proAVP and POMC, respectively ^39,40^. POMC neuron-specific *Sel1L* deletion leads to hyperphagia and age-associated obesity starting around 13 weeks of age when fed a low-fat chow diet ^39^. Given the importance of POMC neurons in maintaining energy homeostasis under various nutritional status, one outstanding question is the relevance and significance of SEL1L-HRD1 ERAD in POMC neurons under pathophysiological conditions, including DIO.

Here, we show that SEL1L-HRD1 ERAD in POMC neurons at the arcuate nucleus (ARC) of the hypothalamus, a key group of metabolic neurons that control food intake and energy expenditure ^43^, controls DIO pathogenesis and leptin sensitivity via the regulation of leptin receptor biogenesis and signaling. Soon after weaning, POMC-specific *Sel1L* deficient (*Sel1L*^*POMC*^) mice are hypersensitive to DIO. Much to our surprise, SEL1L-HRD1 ERAD is indispensable for the maturation of nascent leptin receptor to reach to the cell surface. Hence, SEL1L-HRD1 ERAD is a critical regulator of the maturation of leptin receptor in the ER and thereby leptin signaling in POMC neurons.

## RESULTS

### Transient upregulation of SEL1L-HRD1 ERAD expression in the hypothalamus in response to high fat diet (HFD) feeding.

**Fig. 1:**
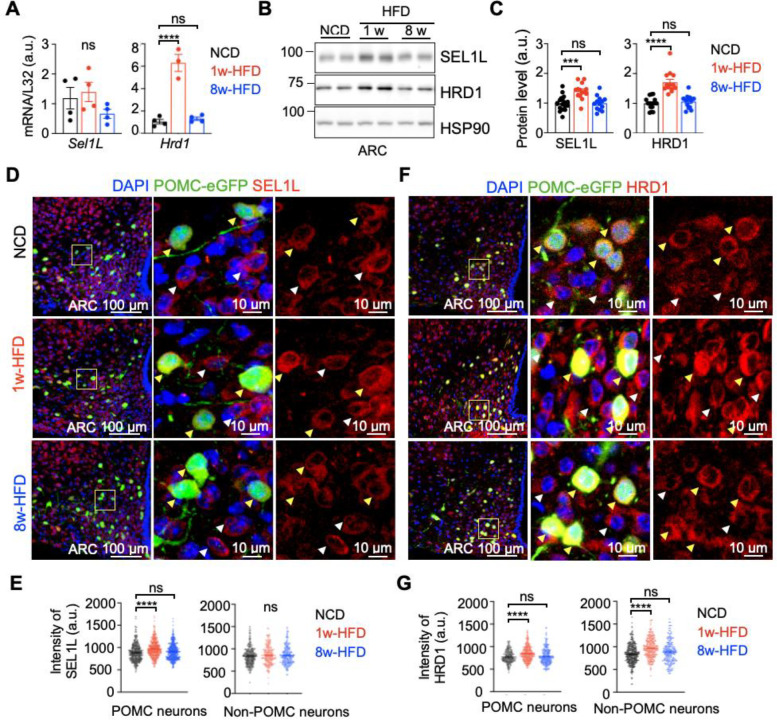
Transient upregulation of SEL1L-HRD1 ERAD expression in the hypothalamus in response to high fat diet (HFD) feeding. **(A)** Quantitative PCR (qPCR) analysis of *Sel1L* and *Hrd1* mRNA levels in the arcuate nucleus (ARC) of the C57BL/6J male mice fed on normal chow diet (NCD), 1w- and 8w-HFD (n=3–4 mice per group). **(B-C)** Representative Western blot of SEL1L and HRD1 in the ARC of the C57BL/6J male mice fed on NCD, 1w- and 8w-HFD, with quantitation shown on the right (n=13–15 mice per group). **(D-E, F-G)** Representative images and quantitation of IF staining of SEL1L (D-E) and HRD1 (F-G) in the ARC of POMC-eGFP mice fed NCD, or HFD for 1-week or 8-week (n=3–4 mice per group, 70–100 POMC and non-POMC cells respectively per mice). Yellow arrows, GFP-positive POMC neurons; White arrows, GFP-negative non-POMC neurons. Values, mean ± SEM. ns., not significant; *p<0.05, **p<0.01, ***p<0.001 and ****p<0.0001 by one-way ANOVA followed by Tukey’s multiple comparisons test (A, C, E, G).

We previously showed that the SEL1L-HRD1 protein complex is constitutively expressed in the ARC of the hypothalamus ^39^. Here we first asked whether its expression in the ARC region is regulated in response to overnutrition by placing the mice on 60% HFD (60% calories derived from fat) for 1 or 8 weeks. HFD feeding expectedly reduced the expression of *Pomc, Npy* and *Agrp* (Supplementary Fig. 1A), while enhance the protein levels of POMC derivatives β-Endorphin and α-MSH (Supplementary Fig. 1B-E). Moreover, HFD feeding enhanced neuronal activity in PVH region as measured by nuclear c-FOS following both 1- and 8-week HFD (Supplementary Fig. 1D, E). One-week HFD significantly induced *Hrd1* mRNA level, but not *Sel1L* mRNA level, while 8-week HFD feeding had no such effect (Fig. 1A). At the protein levels, both SEL1L and HRD1 proteins, were elevated at 1-week HFD, and returned to the basal levels after 8-week HFD (Fig. 1B, C), pointing to the transient response to SEL1L-HRD1 expression in the hypothalamus in response to HFD challenge. We next performed confocal microscopy to visualize SEL1L-HRD1 expression in the ARC regions in response to HFD. To visualize POMC neurons, we used POMC-eGFP transgenic mice where eGFP is under the control of POMC promoter ^39,44^. SEL1L protein level was increased specifically in POMC neurons upon 1-week HFD, and returned to the basal level with prolonged HFD feeding (Fig. 1D, E). Similar observation was obtained for HRD1 protein levels in POMC neurons, but unlike SEL1L, HRD1 protein level was transiently upregulated in non POMC neurons as well (Fig. 1F, G). Hence, SEL1L-HRD1 expression in POMC neurons are responsive to acute, but not chronic, nutrient overload.

### Hypothalamic POMC-specific ERAD deficiency leads to early-set DIO and its pathologies.

**Fig. 2:**
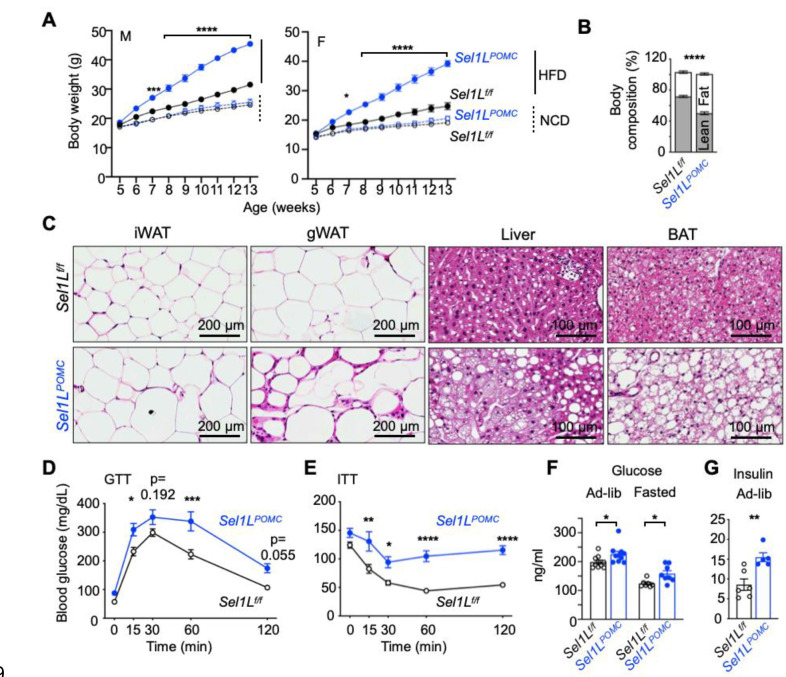
Hypothalamic POMC-specific ERAD deficiency leads to early-set DIO and its pathologies. **(A)** Growth curve of *Sel1L*^*f/f*^ and *Sel1L*^*POMC*^ mice, male (left) and female (right), fed on NCD (open symbols/dotted lines) or HFD (solid symbols/lines) (n=18–24 per group for male mice, n=10–16 per group for female mice). **(B)** Body composition of *Sel1L*^*f/f*^ and *Sel1L*^*POMC*^ male mice after 8w-HFD (n=4–7 mice per group). **(C)** H&E images of peripheral tissues from male mice fed HFD for 8 weeks (n=3 mice per group). iWAT and gWAT, inguinal and gonadal white adipose tissues; BAT, brown adipose tissues. **(D-E)** Glucose tolerance (D) and insulin tolerance tests (E) in male mice fed HFD for 8 weeks. Mice were fasted for 16 or 6 hours prior to glucose (2 g/kg body weight) or insulin (1 unit/kg body weight) injection, respectively (n=6 mice per group). **(F)** Serum glucose in 8w-HFD male mice, either ad-lib or after 6h-fasting (n=7–10 mice per group). **(G)** Insulin levels in 8w-HFD male mice under ad-lib condition (n=5–6 mice per group). Values, mean ± SEM. ns, not significant; *p<0.05, **p<0.01, ***p<0.001 and ****p<0.0001 by two-way ANOVA followed by multiple comparisons test (A-B, D-F) or two-tailed Student’s t-test (G).

To delineate the significance of hypothalamic ERAD in DIO, we next characterized the phenotypes of *Sel1L*^*POMC*^ mice, generated by crossing *Sel1L*^*f/f*^ with the Pomc-Cre mouse line ^39^, following 8-week HFD feeding from 5 weeks of age. While, in line with our previous report, *Sel1L*^*POMC*^ mice appeared comparably to WT littermates in terms of body weight on chow diet for the first 13 weeks of age ^39^ (Fig. 2A), *Sel1L*^*POMC*^ mice, both sexes, gained significantly more body weight soon after HFD feeding (Fig. 2A). Body composition analysis showed that fat content was significantly increased in *Sel1L*^*POMC*^ mice, reaching over 50% of body mass after 8-week HFD (Fig. 2B and Supplementary Fig. 2A) with more lipid deposition in the livers, as well as both white and brown adipose tissues (WAT and BAT) (Fig. 2C). *Sel1L*^*POMC*^ mice became highly glucose intolerant and insulin resistant following 8-week HFD (Fig. 2D, E), with elevated ad libitum and fasting blood glucose (Fig. 2F) and ad libitum insulin levels (Fig. 2G). In addition, glucagon and corticosterone levels were elevated in *Sel1L*^*POMC*^ mice (Supplementary Fig. 2B, C), while rectal temperature in *Sel1L*^*POMC*^ mice was decreased by 2 degrees compared to that of WT littermates (Supplementary Fig. 2D). Hence, we concluded that mice with POMC-specific ERAD defects exhibit early onset DIO and its pathologies including glucose and insulin resistance.

### Hypothalamic ERAD deficiency triggers hyperphagia and leptin resistance.

**Fig. 3:**
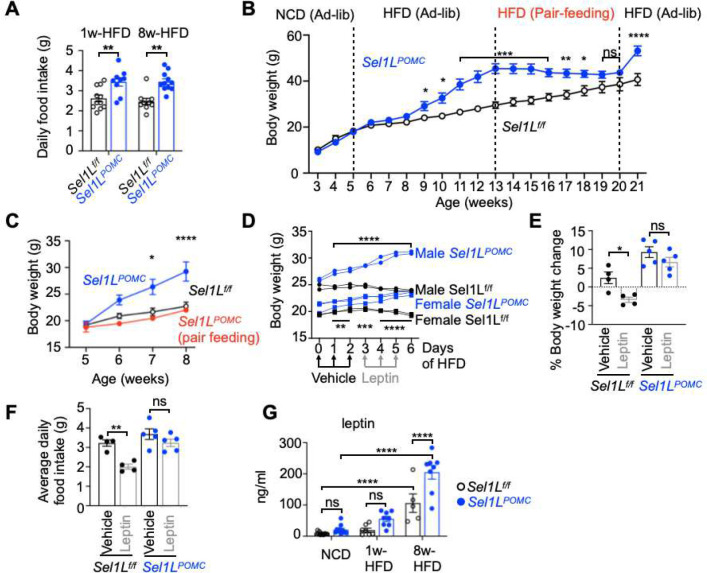
Hypothalamic ERAD deficiency triggers hyperphagia and leptin resistance. **(A)** Daily food intake of male *Sel1L*^*f/f*^ and *Sel1L*^*POMC*^ mice at 1w- and 8w-HFD (n=9–11 mice per group). **(B)** Growth curve of male *Sel1L*^*POMC*^ mice fed with either NCD or HFD under ad libitum or pair feeding as indicated (n=3 mice per group, blue solid circles). Male *Sel1L*^*f/f*^ mice fed ad libitum with the same diets were included as controls (n=3 mice per group, black open circles) **(C)** Growth of *Sel1L*^*POMC*^ male mice with either ad libitum or pair-feeding of HFD starting at 5 weeks of age (n=3–5 mice per group). **(D)** Body weights of 12-week-old mice put on HFD (at day 0) followed by daily i.p. injected with vehicle (PBS) and leptin (2 mg/kg body weight) for 3 days (n=2 per group for male mice, indicated in dots; n=2–3 per group for female mice, indicated in squares). **(E-F)** Percentage of body weight change (E), average daily food intake (F) following 3 daily vehicle and leptin injections of the mice (n=2 per group for male mice, indicated in dots; n=2–3 per group for female mice). % Body weight is calculated based on the body weights at the end point over those at the starting point for each treatment. **(G)** Serum leptin levels in mice fed on NCD, 1w- and 8w-HFD (n=5–13 mice per group). Values, mean ± SEM. ns, not significant; *p<0.05, **p<0.01, ***p<0.001 and ****p<0.0001 by two-way ANOVA followed by multiple comparisons test (A-G).

We next explored the possible mechanism underlying the susceptibility to DIO in *Sel1L*^*POMC*^ mice. *Sel1L*^*POMC*^ mice consumed ~ 40% more food daily, i.e., hyperphagia, upon both 1- and 8-week HFD feeding (Fig. 3A). To directly demonstrate the direct causal link between food intake and weight gain, we performed pair feeding (giving the same amount of the food as WT littermates consume) following 8-week ad libitum HFD feeding. *Sel1L*^*POMC*^ mice gained weight quite rapidly under *ad libitum* feeding of HFD; however, their weight gain was significantly slowed down following pair-feeding and recovered when placed on ad libitum HFD feeding again (Fig. 3B). Indeed, weight gain of *Sel1L*^*POMC*^ mice was comparable to that of WT littermates if pair-feeding was performed at the beginning of HFD feeding (Fig. 3C). We then tested whether hyperphagia of *Sel1L*^*POMC*^ mice is caused by leptin resistance by leptin injection (Fig. 3D). Leptin injection was expected to induce body weight loss in WT mice, but not *Sel1L*^*POMC*^ mice. Indeed, unlike WT mice, *Sel1L*^*POMC*^ mice continued to gain body weight following leptin injection (Fig. 3D, E). This difference in body weight gain was likely due to the differences in food intake in response to leptin injection (Fig. 3F), pointing to a significant leptin resistance in *Sel1L*^*POMC*^ mice. *Sel1L*^*POMC*^ mice exhibited progressively marked hyperleptinemia with HFD feeding (Fig. 3G). Hence, we concluded that hypothalamic POMC neurons-specific ERAD deficiency triggers hyperphagia and leptin resistance.

### The effect of hypothalamic SEL1L-HRD1 ERAD in DIO is mediated by leptin resistance.

**Fig. 4:**
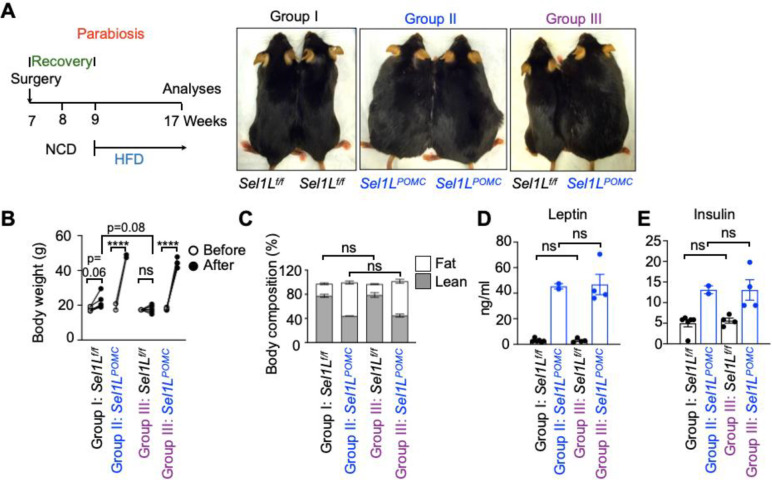
Hypothalamic SEL1L-HRD1 deficiency leads to DIO via leptin signaling. **(A)** Schematic diagram for parabiosis and pictures (right) of *Sel1L*^*f/f*^ and *Sel1L*^*POMC*^ female mice after parabiosis HFD for 8 weeks (n=3 pairs in group I, n=1 pair in group II, n=5 pairs in group III). **(B-C)** Body weights (B) of mice before and after parabiosis and body composition (C) after parabiosis following 8-week HFD for 8 weeks (n=6 mice in group I, n=2 mice in group II, n=5 mice per genotype in group III). **(D-E)** Serum leptin (D) and insulin (E) levels of mice after parabiosis HFD for 8 weeks (n=6 mice in group I, n=2 mice in group II, n=5 mice per genotype in group III). Values, mean ± SEM. ns, not significant; *p<0.05, **p<0.01, ***p<0.001 and ****p<0.0001 by two-way ANOVA followed by multiple comparisons test (B-E).

To further establish the effect of leptin resistance in ERAD deficiency-associated DIO, we next performed parabiosis where two littermates were surgically stitched together to allow the sharing of the circulation (Fig. 4A). Following two weeks of recovery on chow diet, the parabionts *WT*: *Sel1L*^*POMC*^ (Group III) were placed on HFD for 8 weeks (Fig. 4A). Two control parabionts, *WT: WT* (Group I) and *Sel1L*^*POMC*^:*Sel1L*^*POMC*^ (Group II), gained weight as expected with the latter pair becoming obese (Fig. 4B). However, in *WT*: *Sel1L*^*POMC*^ (Group III) parabionts, body weight gain for WT mice was attenuated compared to WT mice in *WT: WT* (Group I) control parabionts (P=0.08), while body weight gain for *Sel1L*^*POMC*^ mice was comparable to that of *Sel1L*^*POMC*^:*Sel1L*^*POMC*^ parabionts (Group II) (Fig. 4B). Body compositions (i.e., lean vs. fat) in parabionts were not affected by the partner (Fig. 4C). Moreover, serum leptin and insulin levels were highly elevated in the *Sel1L*^*POMC*^ mice, but unaltered in WT mice regardless of the partners (Fig. 4D, E). Hence, these data suggested that hypothalamic SEL1L-HRD1 ERAD controls DIO pathogenesis via hyperleptinemia.

### Hypothalamic SEL1L-HRD1 ERAD deficiency impairs leptin-pSTAT3 signaling.

**Fig. 5:**
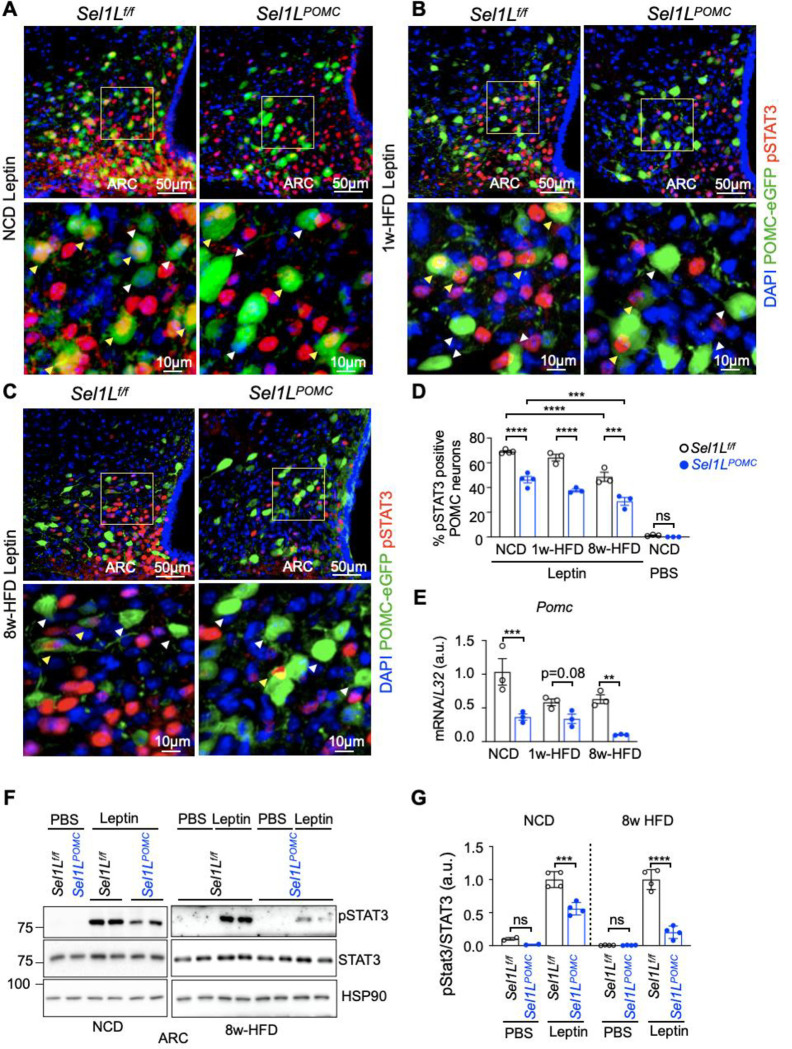
Hypothalamic SEL1L-HRD1 ERAD deficiency impairs leptin-pSTAT3 signaling. **(A-D)** Representative immunofluorescence (IF) staining of pSTAT3 in *Sel1L*^*f/f*^;POMC-eGFP and *Sel1L*^*POMC*^;POMC-eGFP mice at NCD (A), 1w-HFD (B) and 8w-HFD (C), with quantitation shown in D. Mice were fasted for overnight (16hrs) and administrated with leptin (i.p., 2 mg/kg body weight) for 30 min (n=3–4 mice per group). Yellow arrows, pSTAT3 positive POMC neurons; White arrows, pSTAT3 negative POMC neurons. PBS-injected mice were included as negative controls and shown in Supplementary Fig. 3. **(E)** Quantitative PCR (qPCR) analysis of *Pomc* mRNA expression levels in ARC of *Sel1L*^*f/f*^ and *Sel1L*^*POMC*^ mice at 8w-HFD (n=3 mice per group). **(F-G)** Representative Western blot for pSTAT3 in ARC of *Sel1L*^*f/f*^ and *Sel1L*^*POMC*^ mice at NCD or 8w-HFD, injected with leptin or PBS for 30 min (n=4 male mice per group), with quantitation shown in G. Values, mean ± SEM. ns, not significant; *p<0.05, **p<0.01, ***p<0.001 and ****p<0.0001 by two-way ANOVA followed by multiple comparisons test (D, E, G).

We next asked how POMC-specific SEL1L-HRD1 ERAD regulates leptin sensitivity. As leptin signaling induces phosphorylation of STAT3 (pSTAT3), we next examined the levels of pSTAT3 in POMC neurons following leptin challenge. To visualize POMC neurons, we generated *Sel1L*^*POMC*^ mice on the POMC-eGFP background (*Sel1L*^*POMC*^;POMC-eGFP) ^39,44^. HFD feeding progressively blunted leptin-induced pSTAT3 in the POMC neurons of the ARC region of WT mice, but to a much greater extent, in *Sel1L*^*POMC*^ mice (Fig. 5A–D and Supplementary Fig. 3). In keeping with the notion that pSTAT3 a critical transcription factor for the *Pomc* gene ^45^, hypothalamic *Pomc* mRNA expression was markedly decreased in *Sel1L*^*POMC*^ mice with HFD (Fig. 5E). Moreover, Western blot analysis of pSTAT3 of the ARC region also showed a greater reduction of the percent of STAT3 being phosphorylated following HFD feeding (Fig. 5F, G). Thus, our data suggested that SEL1L-HRD1 ERAD in POMC neurons is vital for maintaining central leptin sensitivity during DIO pathogenesis.

### The effect of POMC-specific ERAD in DIO is likely uncoupled from UPR or inflammation.

**Fig. 6:**
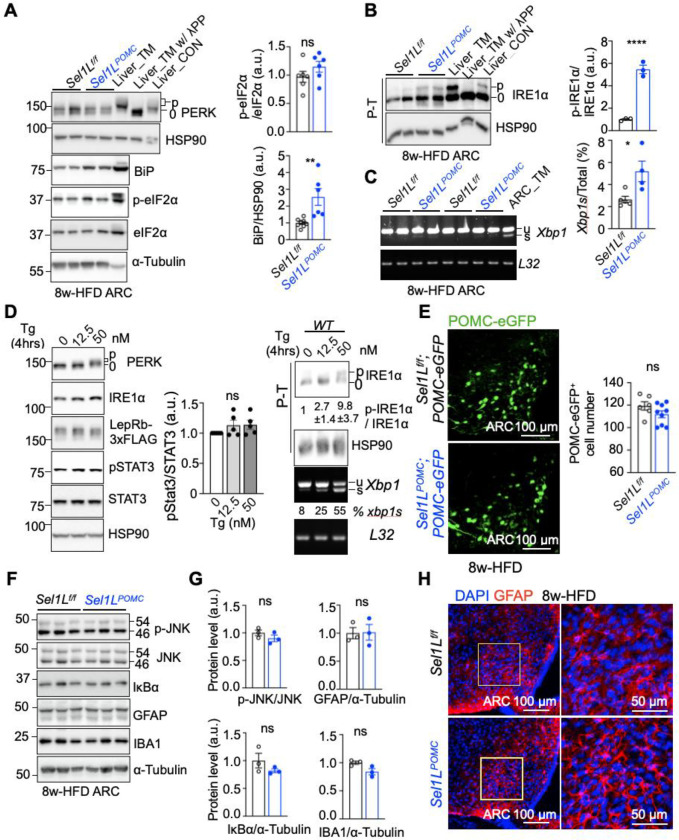
The effect of POMC-specific ERAD in DIO is likely uncoupled from UPR and inflammation. **(A)** Representative Western blot for the PERK pathway of UPR in the ARC of *Sel1L*^*f/f*^ and *Sel1L*^*POMC*^ mice fed on 8w-HFD (n=6 mice per group with 3 male mice and 3 female), with quantitation shown on the right. Livers of mice treated with tunicamycin (TM, 1 mg/kg, i.p.) for 24 hours (Liver_TM) or not (Liver_CON), as well as lysates treated with Lambda protein phosphatase, included as controls. **(B)** Phostag gel (P-T)-based Western blot for IRE1α phosphorylation in the ARC of *Sel1L*^*f/f*^ and *Sel1L*^*POMC*^ mice fed on 8w-HFD, with quantitation shown on the right (n=3 mice per group with 2 male and 1 female). **(C)** Reverse transcriptase PCR (RT-PCR) analysis of *Xbp1* mRNA splicing (u, unspliced; s, spliced) in ARC of *Sel1L*^*f/f*^ and *Sel1L*^*POMC*^ mice fed on 8w-HFD (n=2–3 male mice and n=2–3 female mice per group), with quantitation shown on the right. ARC of mice treated with tunicamycin (TM, 1 mg/kg, i.p.) for 24 hours (ARC_TM) included as a positive control. **(D)** Representative assays for UPR and pSTAT3 in mLepRb-transfected HEK293T treated with leptin with/without Thapsigargin (Tg) (n=5 independent cell samples for SDS-PAGE gel, n=3 for P-T gel, two independent repeats for RT-PCR). **(E)** Representative confocal images of the number of GFP-expressing POMC neurons in *Sel1L*^*f/f*^*;*POMC-eGFP and *Sel1L*^*POMC*^*;*POMC-eGFP mice after 8w-HFD, with quantitation shown on the right (n=6–9 mice per group). **(F-G)** Representative Western blot analysis of inflammatory markers in the ARC of *Sel1L*^*f/f*^ and *Sel1L*^*POMC*^ mice fed on 8w-HFD, with quantitation shown in G (n=3 mice per group). **(H)** Representative confocal images of GFAP, a marker of astrocytes, in the ARC of male *Sel1L*^*f/f*^ and *Sel1L*^*POMC*^ mice fed on 8w-HFD (n=3 mice per group). Values, mean ± SEM. ns, not significant; *p<0.05, **p<0.01, ***p<0.001 and ****p<0.0001 by two-way ANOVA followed by multiple comparisons test (D) or two-tailed Student’s t-test (A-C, E, G).

As ERAD deficiency expectedly causes the accumulation of unfolded/misfolded proteins in the ER that can potentially trigger UPR and given the reported role of UPR in DIO pathogenesis, we next tested whether ERAD deficiency activates UPR and if so, to what extent. There was no detectable activation of the PERK pathway as measured by phosphorylation of PERK and its downstream phosphorylation of eIF2α (Fig. 6A and Supplementary Fig. 4A). Phosphorylation of IRE1α, on the other hand, was moderately elevated in the ARC of *Sel1L*^*POMC*^ mice, so was the splicing of *Xbp1* mRNA (a downstream effector of IRE1α) (Fig. 6B, C and Supplementary Fig. 4B, C). Consistently, ER chaperons BiP (an XBP1 target) was mildly elevated in the ARC of *Sel1L*^*POMC*^ mice (Fig. 6A and Supplementary Fig. 4A, D). In vitro, treatment with an ER stress inducer thapsigargin (Tg) induced strong ER stress, but failed to affect leptin signaling in WT HEK293T cells transfected with long isoform of mouse Leptin receptors (mLepRb) (Fig. 6D and Supplementary Fig. 4E), indicating that UPR is not sufficient to induce leptin resistance. Importantly, we found no significant POMC neuronal loss in the ARC of *Sel1L*^*POMC*^;POMC-eGFP mice (Fig. 6E). Inflammatory markers were largely comparable in the ARC of *Sel1L*^*POMC*^ mice compared to those in WT littermates as measured by phosphorylation and protein levels of c-Jun N-terminal Kinase (JNK) as well as protein levels of I kappa B alpha (IκBα) (Fig. 6F, G). Chronic HFD feeding mildly increased astrogliosis in the ARC regions of both *Sel1L*^*POMC*^ and *Sel1L*^*POMC*^ mice as measured by both Western blot and immunofluorescence staining of astrocyte marker Glial Fibrillary acidic protein (GFAP) and/or microglia marker Ionized calcium-binding adaptor molecule 1 (IBA1) (Fig. 6F–H and Supplementary Fig. 4F). Taken together, these data demonstrate that *Sel1L* deficiency in POMC neurons triggers leptin resistance, independently of UPR, neuronal cell death and inflammation.

### SEL1L-HRD1 is required for the maturation of nascent leptin receptor (LepR).

**Fig. 7:**
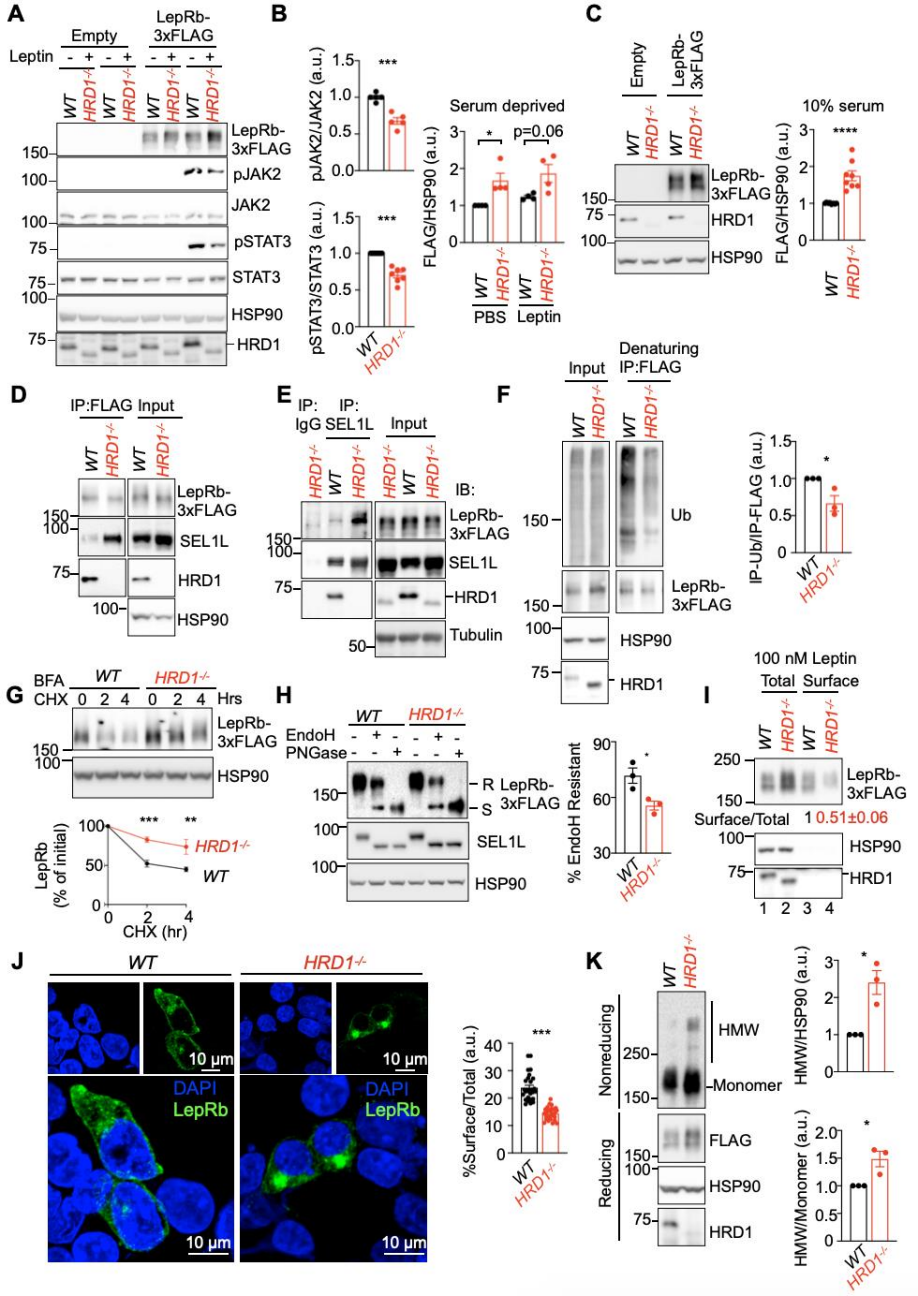
SEL1L-HRD1 is required for the maturation of nascent LepRb. **(A-B)** Representative Western blot analysis for pJAK2, pSTAT3 and LepRb in HEK293T transfected with or without mLepR, treated with or without leptin (A), with quantitation shown in (B) (n=4–7 individual cell samples per group). **(C)** Representative Western blot analysis of mLepRb protein levels in mLepRb-transfected HEK293T in complete medium (DMEM w/ 10% FBS), with quantitation shown on the right (n=8 individual cell samples per group). **(D-E)** Representative Western blot analysis of interaction between SEL1L-HRD and mLepRb following immunoprecipitation (IP) of Flag (D) or SEL1L (E) from lysates of mLepRb-transfected HEK293T (n=2–3 individual cell samples). **(F)** Representative Western blot analysis of Ub following denaturing immunoprecipitation (IP) of Flag from lysates of mLepRb-transfected HEK293T, with quantitation shown on the right (n=3 individual cell samples per group). **(G)** Representative Western blot analysis of LepRb protein decay in LepRb-transfected HEK293T cells co-treated with protein trafficking inhibitor Brefeldin-A and translation inhibitor cycloheximide (CHX) for the 0, 2 and 4 hours, with quantitation shown below (n=4 individual cell samples per group). **(H)** Representative Western blot analysis of LepRb glycosylation in LepRb-transfected HEK293T with EndoH and PNGase treatment, with quantitation shown on the right (n=3 individual cell samples per group). **(I)** Representative Western blot analysis of mLepRb membrane display by surface biotinylation and streptavidin-bead pull down assay in mLepRb-transfected HEK293T treated with leptin. T, total lysate; S, surface fraction. (n=2 individual cell samples per group). **(J)** Representative IF images of LepRb in mLepRb-transfected HEK293T treated with leptin, with quantitation of %surface signals over total shown on the right (n=28 cells per genotype from 3 independent repeats). **(K)** Reducing and non-reducing SDS-PAGE and Western blot analysis of LepRb high molecular-weight aggregates of LepRb in mLepRb-transfected WT and HRD1^−/−^ HEK293T, with quantitation shown on the right (n=3 individual cell samples per group). Values, mean ± SEM. ns, not significant; *p<0.05, **p<0.01, ***p<0.001 and ****p<0.0001 by two-tailed Student’s t-test (A, C, F, H, J, K) or two-way ANOVA followed by multiple comparisons test (B, G).

The forementioned data suggested that SEL1L-HRD1 ERAD regulates leptin sensitivity upstream of STAT3. To further explore the underlying mechanism, we generated leptin-responsive HEK293T cell system expressing the long isoform of LepR (LepRb) responsible for leptin-induced JAK2-STAT3 signaling ^46–49^. Indeed, in line with decreased leptin sensitivity in vivo, mLepRb-positive *HRD1*^*−/−*^ HEK293T cells exhibited impaired phosphorylation of JAK2 and STAT3 compared to those in transfected *WT* cells in response to leptin stimulation (Fig. 7A, B). Surprisingly, the protein level of mLepRb was significantly higher in *HRD1*^*−/−*^ HEK293T cells compared to that of WT cells, under both serum-deprived and -supplemented conditions (Fig. 7B, C). Moreover, SEL1L interaction with in LepRb-transfected cells was markedly enhanced in *HRD1*^*−/−*^ cells where substrate-SEL1L interaction is known to be stabilized ^29,50,51^ (Fig. 7D, E). LepRb was ubiquitinated in an HRD1-dependent manner (Fig. 7F) and was significantly stabilized in *HRD1*^*−/−*^ cells compared to that in *WT* cells (Fig. 7G).

We next assess the consequence of ERAD deficiency on LepRb maturation in the ER. Endoglycosidase H (EndoH) digestion, which cleaves asparagine-linked high mannose or hybrid glycans of the immature glycoproteins predominantly in ER ^52^, revealed significantly lower fraction of EndoH resistant form of LepRb that were able to exit the ER for complete maturation in *HRD1*^*−/−*^ HEK293T cells (Fig. 7H). This was further confirmed by surface biotinylation assay followed by immunoprecipitation with streptavidin-beads, which indicated reduced proportion of surface LepRb in *HRD1*^*−/−*^ cells (Fig. 7I and Supplementary Fig. 5A). Moreover, confocal microscopy following immunofluorescence staining further demonstrated an altered distribution of LepRb with increased intracellular, but decreased surface, expression in ERAD-deficient cells (Fig. 7J and Supplementary Fig. 5B). In the absence of SEL1L-HRD1, LepRb protein was prone to form high molecular weight aggregates via disulfide bonds (Fig. 7K) Taken together, our data show that SEL1L-HRD1 ERAD is required for the maturation of LepRb by targeting the misfolding-prone or misfolded LepRb for proteasomal degradation.

### SEL1L-HRD1 ERAD degrades and limits the pathogenicity of human LepRb Cys604Ser (C604S) mutant.

**Fig. 8:**
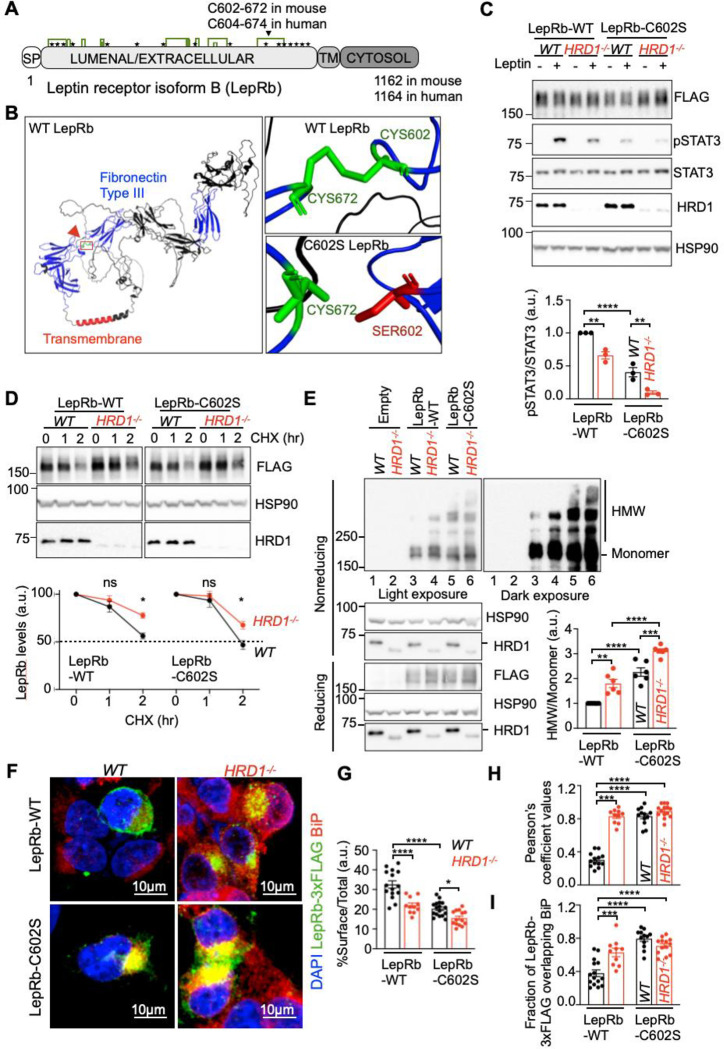
SEL1L-HRD1 ERAD degrades and limits the pathogenicity of LepRb Cys602Ser disease mutant. **(A)** Schematic diagram of mouse LepRb. “SP”, Signal Peptide; “TM”, Transmembrane. Star symbols, N-glycosylation sites; Green lines, disulfide bonds. **(B)** Structural modeling of mouse LepRb by AlphaFold2. Red arrow, location of human mutation C604S (mouse C602S). **(C)** Representative Western blot analysis for pSTAT3 in HEK293T transfected with mLepRb-WT or mLepRb-C602S with or without leptin treatment, with quantitation shown below (n=3 individual cell samples per group). **(D)** Representative Western blot analysis of LepRb protein decay in WT and HRD1^−/−^ HEK293T transfected with mLepRb-WT or -C602S, treated with brefeldin-A and cycloheximide (CHX) for the 0, 1 and 2 hours, with quantitation shown below (n=4 individual cell samples per group). **(E)** Reducing and non-reducing SDS-PAGE and Western blot analysis of LepRb high molecular-weight (HMW) aggregates of LepRb in WT and HRD1^−/−^ HEK293T transfected with mLepRb-WT or -C602S, with quantitation shown on the right (n=6 individual cell samples per group). **(F-I)** Representative IF images of mLepRb-WT and -C602S in transfected WT and HRD1^−/−^ HEK293T cells (F) with quantitation %surface signals over total (G) (n=11–17 cells per group) and analysis of co-localization of LepRb with BiP signals by Pearson correlation coefficient (H) and Manders overlap coefficient (I) (n=10–14 cells per group). Values, mean ± SEM. ns, not significant; *p<0.05, **p<0.01, ***p<0.001 and ****p<0.0001 by two-way ANOVA followed by multiple comparisons test (C, D, E, G, H, I).

To demonstrate the clinical relevance of our findings, we asked whether human LepRb (hLepRb) mutants ^53,54^ are SEL1L-HRD1 ERAD substrates. Here, we focused on hLepRb mutant C604S, a recessive point mutation due to missense homozygous substitution T > A at position 1810, identified in two brothers at 1- and 5-years old with severely early onset obesity ^54,55^. C604-C674 forms a disulfide bond in human LepRb corresponding to C602-C672 in mouse LepRb (Fig. 8A, B) ^56–58^. This mutation has been predicted as loss-of-function likely due to defects in folding ^54,56–58^. C602S mLepRb significantly impaired leptin response compared to WT mLepRb in *WT* cells, which was further diminished in *HRD1*^*−/−*^ cells (Fig. 8C). Similar to WT mLepRb, C602S mLepRb was stabilized in the absence of HRD1 (Fig. 8D). Notably, C602S mLepRb readily formed HMW aggregates in *WT* HEK293T cells, and to much greater extent, in *HRD1*^*−/−*^ cells (Fig. 8E). Such aggregates likely formed in the ER as demonstrated by their colocalization with the ER chaperone BiP based on immunostaining (Fig. 8F–I). Hence, SEL1L-HRD1 ERAD is indispensable for the degradation of nascent WT and, at least a subset of, disease mutant LepRb, which ensures the maturation, trafficking and membrane display of functional LepRb.

## DISCUSSION

**Fig. 9:**
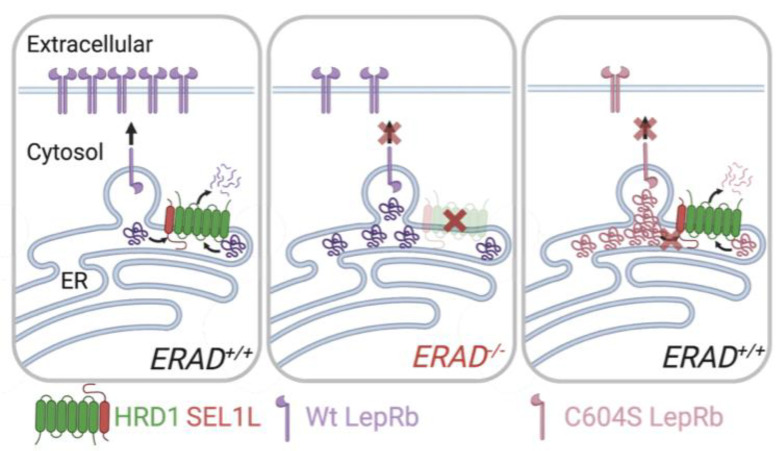
Proposed models for SEL1L-HRD1 ERAD degradation of wildtype LepRb and C604S disease mutant. In the basal conditions, SEL1L-HRD1 ERAD constitutively degrades misfolded LepRb and ensures the proper folding, maturation and surface expression of the LepRb. In the absence of ERAD, the accumulation of misfolded receptors forms aggregates, interferes with the folding and maturation of the nascent LepRb with attenuated surface display. In the context of recessive LepRb C604S mutant, though degraded by SEL1L-HRD1 ERAD, C604S LepRb readily forms aggregates to the extent beyond the capacity of ERAD, resulting in impaired maturation and surface display of the receptors.

This study not only identifies a novel regulatory mechanism for leptin receptor and signaling, but also reports a key role of hypothalamic ERAD in maintaining energy homeostasis under nutrient overload conditions. SEL1L-HRD1 ERAD defects in POMC neurons predispose mice to DIO and its pathologies, due to hyperphagia and hypothalamic leptin resistance. Our mechanistic studies establish LepRb as a bona fide endogenous substrate of SEL1L-HRD1 ERAD. Pointing to the clinical relevance of our findings, human recessive LepRb C604S variant is trapped in the ER and degraded by SEL1L-HRD1 ERAD (Fig. 9). In the absence of SEL1L-HRD1 ERAD, both WT and C604S LepRb are trapped in the ER in the form of HMW aggregates, with attenuated cell surface expression (Fig. 8E–I and Fig. 9). While this reported effect of ERAD in POMC neurons is in keeping with recent studies demonstrating the profound physiological importance of SEL1L-HRD1 ERAD in vivo ^39,40^, it uncovers a novel function of SEL1L-HRD1 ERAD in leptin signaling and a novel regulatory mechanism for leptin biology.

Our data show that hypothalamic SEL1L deficiency markedly increases the progression and pathogenesis of DIO in mice. *Sel1L*-deficient POMC neurons exhibit mild alterations in ER homeostasis including elevated activation of the IRE1α -XBP1 pathway and expression of ER chaperones, but without any detectable cell death. As previous studies have shown that deficiency of *Ire1a* or *Xbp1* in POMC neurons predispose mice to DIO ^21^, while gain-of-function of XBP1s in POMC neurons had an opposite effect ^23^, we conclude that the effect of SEL1L-HRD1 ERAD is uncoupled from IRE1α -XBP1 pathway of the UPR and cell death, which is in line with many recent studies of various tissue-specific *Sel1L*- or *Hrd1*-deficient models ^37,39–42,59^. These findings point to the cellular adaption in response to ERAD deficiency ^25^. Such mild UPR activation and chaperone expression are potentially cyto-protective in response to the accumulation of misfolding proteins in the ER.

Previous reports have suggested that UPR may play a causal role in leptin resistance due to impaired leptin signaling ^15,17,60^. These studies were performed via the administration of ER stress inducers tunicamycin and thapsigargin which can be fraught with artefacts. Indeed, tunicamycin can inhibit glycosylation of the glycoproteins^61^ including LepRb, and thus the impaired leptin signaling can be directly due to defective glycosylation and concomitant functionality of LepRb instead of UPR activation as a general outcome of numerous dysregulation of glycoproteins. Further, high dosage of ER stress inducers included in previous studies may fall far from any physiological relevance^15,17,60^. In our study, thapsigargin treatment induced a range of ER stress response in a dose dependent manner, but failed to alter leptin signaling in *WT* HEK293T cells transfected with mLepRb even at the high level of UPR. Hence, collective evidence suggests that UPR is likely uncoupled from leptin signaling. The reason for these discrepancies remains unknown. Careful future studies are needed to validate either model.

This study demonstrates an important role of SEL1L-HRD1 ERAD in leptin signaling, at least in part via the regulation of the maturation of nascent LepRb protein. We previously showed that SEL1L-HRD1 ERAD is required for the posttranslational maturation of POMC prohormone in mice on chow diet and that *Sel1L* deficiency in POMC neurons cause age-associate obesity in mice on chow diet due to the ER retention of POMC prohormone ^39^. In DIO mouse models, we found defects in *Sel1L*^*POMC*^ mice occurring upstream of POMC transcription as leptin-induced STAT3 phosphorylation is impaired in the absence of SEL1L-HRD1 ERAD ^45–49^. Further mechanistic studies identify partial loss-of-function of LepRb resulted from attenuated ER exit of nascent LepRb in SEL1L-HRD1 ERAD deficient cells. This study suggests that nascent LepRb protein is likely misfolding prone in the ER, likely due to multiple glycosylation and the formation of disulfide bonds, and hence relies on SEL1L-HRD1 ERAD to generate an ER environment conducive for the proper folding and conformation of bystander LepRb.

Several human mutants have also been identified as SEL1L-HRD1 ERAD substrates that readily form aggregates and become resistant to and bypassing the quality control mediated by ERAD, leading to loss-of-function disease phenotype. These misfolded substrates with highly reactive cysteine thiols accumulate and promote the formation of inter- or intra-molecular disulfide-bonded aggregates ^39–41^. Hence, SEL1L-HRD1 ERAD-mediated degradation of nascent unfolded and misfolded substrates, including LepRb in this study, may effectively prevent protein aggregation and maintain the folding environment in the ER. Efforts to target SEL1L-HRD1 ERAD function may represent a viable means for the treatment of certain diseases caused by a dominant-negative disease allele or a general collapse of the folding environment in the ER.

## METHODS

### Mice.

As described previously ^39^, POMC-specific *Sel1L*-deficient mice (*Sel1L*^*POMC*^) and control littermates (*Sel1L*^*f/f*^) were generated. The mice were further crossed with Pomc-eGFP reporter mice to generate *Sel1L*^POMC^;POMC-eGFP and control littermates *Sel1L*^*f/f*^;POMC-eGFP. WT B6 mice were purchased from JAX and bred in our mouse facility. Mice were fed a chow diet (13% fat, 57% carbohydrate and 30% protein, PicoLab Rodent Diet 5L0D) and placed on a high-fat diet (HFD, calories provided by 60% fat, 20% carbohydrate and 20% protein, Research Diet D12492) from 5 weeks of age for 1 week or 8 weeks. All mice were housed in a temperature-controlled room with a 12-hour light/12-hour dark cycle.

### Food intake measurement and pair-feeding.

Food intake were measured as previously described ^39^. Briefly, to perform daily food intake measurement, mice were first acclimatized to single housing 24 hours before the experiment. Daily food intake was measured 1 hour before the onset of the dark cycle each day. For the pair-feeding at later stage of HFD feeding, *Sel1L*^*POMC*^ and WT littermates had continuous free access to HFD for eight weeks and were then single housed and fed ~2.5 g, which was determined by the average of daily food intake of WT littermates, at the start of the dark cycle. For the pair-feeding at early stage of HFD feeding, 5-week-old *Sel1L*^*POMC*^ mice were split into two groups: One group of *Sel1L*^*POMC*^ and WT littermates had continuous free access to food; the other group of *Sel1L*^*POMC*^ mice (pair-fed) was fed ~2.5 g at the start of dark hours. Weekly bodyweight gains were monitored.

### Leptin treatment in mice.

Twelve-week-old mice were intraperitoneally (i.p.) injected PBS followed by leptin (2 mg/kg body weight, R&D systems; catalog 498-OB-05M) 1 hour before the onset of dark cycle for three consecutive days as described ^39^. Body weight and food intake were monitored daily during the treatment period. For phosphorylated STAT3 staining, 2 mg/kg leptin were i.p. injected to mice, followed by overnight fasting. Mice were anesthetized by isoflurane for fixation-perfusion 30 min after injection.

### Tissue and blood collection.

These procedures were carried out as previously described^39^. Briefly, blood was collected from anesthetized mice via cardiac puncture, transferred to 1.5ml microcentrifuge tubes, kept at room temperature for 30 minutes prior to centrifugation at 2,000 *g* for 15 minutes. Serum was aliquoted and stored at −80°C until analysis. For brain microdissection, Adult Mouse Brain Slicer Matrix (BSMAA001–1, Zivic Instruments) was used to collect coronal brain slices containing ARC region with further microdissection to obtain ARC-enriched region. All tissues were snap-frozen in liquid nitrogen and stored at −80°C before use.

### Preparation of brain sections.

Mice were anesthetized with isoflurane, perfused with PBS followed by 4% paraformaldehyde (PFA) (Electron Microscopy Sciences; catalog 19210) for fixation. Brains were then postfixed in 4% PFA for overnight at 4°C, dehydrated in 15% sucrose and then 30% sucrose consecutively overnights at 4°C, and sectioned (30 μm) on a cryostat (Microm HM550 Cryostat, Thermo Fisher Scientific). The sections were stored in DEPC-containing anti-freezing media (50% 0.05 M sodium phosphate pH 7.3, 30% ethylene glycol, 20% glycerol) at −20°C. Different brain regions were identified using the Paxinos and Franklin atlas. Counted as distance from bregma, the following coordinates were used: PVN (−0.82 mm to −0.94 mm) and ARC (−1.58 mm to −1.7 mm).

### Western blot and antibodies.

Frozen tissue or cells were homogenized by sonication in lysis buffer [150mM NaCl, 50mM Tris pH 7.5, 10 mM EDTA, 1% Triton X-100] with freshly added protease inhibitors (Sigma; catalog P8340), phosphatase inhibitors (Sigma; catalog P5726) and 10 mM N-ethylmaleimide (Thermo Scientific; catalog 23030). Lysates were incubated on ice for 30 min followed by centrifugation (13,000 g, 10 min at 4 °C). Supernatants were collected and analyzed for protein concentration using Bradford assay (Bio-Rad; catalog 5000006). For denaturing SDS-PAGE, samples were further suppled with 1mM DTT and denatured at 95°C for 5 min in 5x SDS sample buffer (250 mM Tris-HCl pH 6.8, 10% sodium dodecyl sulfate, 0.05% Bromophenol blue, 50% glycerol, and 1.44 M β-mercaptoethanol). For non-reducing SDA-PAGE, samples were prepared in 5x non-denaturing sample buffer (250 mM Tris-HCl pH 6.8, 10% sodium dodecyl sulfate, 0.05% bromophenol blue, 50% glycerol). For phostag gel analysis based on phos-tag system as described^62,63^, SDS-PAGE gel was supplemented by 50μM MnCl2 (Sigma) and 25μM phostag reagent (NARD Institute; catalog AAL-107) and must be protected from light until finishing running. Protein isolated from the liver of mice treated with tunicamycin (TM, 1 mg/kg, i.p.) for 24 hours was used as a positive control to indicate the position of phosphorylated PERK and IRE1a. For phosphatase treatment, 100 μg tissue lysates were incubated with 1 μl lambda phosphatase (λPPase, New England BioLabs; catalog P0753S) in 1× PMP buffer (New England BioLabs; catalog B0761S) with 1 mM MnCl_2_ (New England BioLabs; catalog B1761S) at 30°C for 30 min. Reaction was stopped by adding 5× SDS sample buffer and incubated at 90°C for 5 min.

All samples were incubated in 65°C for 10min and run with 15–30 μg total lysate on SDS-PAGE gel for separation followed by electrophoretic transfer to PVDF membrane (0.45μm, Millipore; catalog IPFL00010). The blots were incubated in 2% BSA/Tri-buffered saline tween-20 (TBST) with primary antibodies overnight at 4°C, washed with TBST followed by 1hr incubation with goat anti-rabbit or mouse IgG HRP at room temperature. Band density was quantitated using the Image Lab software on the ChemiDOC XRS+ system (Bio-Rad).

Antibodies for Western blot were as follows: SEL1L (rabbit, 1:8000, Abclonal; catalog E112049), HRD1 (rabbit, 1:2000, ABclonal; catalog E15102), GRP78 BiP (rabbit, 1:5000, Abcam; catalog ab21685), HSP90 (rabbit, 1:5,000, Santa Cruz Biotechnology Inc.; catalog sc-7947), FLAG (mouse, 1:2000, Sigma-Aldrich; catalog F-1804), IRE1α (rabbit, 1:2,000, Cell Signaling Technology; catalog 3294), p-eIF2α (rabbit, 1:2000, Cell Signaling Technology; catalog 3597), eIF2α (rabbit, 1:2000, Cell Signaling Technology; catalog 9722), p-JNK (mouse, 1:2000, Cell Signaling Technology; catalog 9255), JNK (rabbit, 1:1000, Cell Signaling Technology; catalog 9252), PERK (Rabbit, 1:1000, Cell Signaling Technology; catalog 3192), pSTAT3 (Tyr705) (rabbit, 1:1000, catalog 9131, Cell Signaling Technology), STAT3 (rabbit, 1:1000, Cell Signaling Technology; catalog 9132), pJAK2 (Tyr1007/1008) (rabbit, 1:1000, Cell Signaling Technology; catalog 3771), JAK2 (rabbit, 1:1000, ABclonal; catalog A19629), Tubulin (mouse, 1:5000, Santa Cruz Biotechnology Inc.; catalog sc-5286), IκBα (rabbit, 1:1000, Cell Signaling Technology; catalog 9242) and IBA1 (rabbit, 1:1000, Proteintech; catalog 10904–1-AP) Secondary antibodies for Western blot were goat anti-rabbit IgG HRP and goat anti-mouse IgG HRP at 1:5,000, both from Bio-Rad.

### Immunostaining and antibodies.

For fluorescent immunostaining in free-floating brain sections, samples were picked out of anti-freezing buffer followed by 3 washes with PBS. Free-floating sections were simultaneously incubated with primary antibodies in blocking buffer (0.3% donkey serum and 0.25% Triton X-100 in 0.1 M PBS) overnight at 4°C. Following 3 washes with PBS, sections were incubated with secondary antibodies for 2 hours at room temperature. Brain sections were then mounted on gelatin-coated slides (Southern Biotech; catalog SLD01-CS). Counterstaining and mounting were performed with mounting medium containing DAPI (Vector Laboratories; catalog H-1200) and Fisherfinest Premium Cover Glasses (Fisher Scientific; catalog 12–548-5P). For immunostaining in cells, 24 hours after transfection of LepRb-3xFLAG constructs, cells were placed on Poly-L-Lysine (Advanced Biomatrix; catalog 5048) coated Millicell EZ SLIDE 8-well glasses (Millipore; catalog PEZGS0816) for 24 hours before treatment and fixation. For staining surface bound leptin, samples were washed by ice cold PBS for 5 times and fixed by 4% formaldehyde (VWR; catalog 89370–094) for 15 minutes on ice followed by 3 washes with PBS. No permeabilization reagents were involved. For staining other markers, permeabilization was included and the overall process were the same as described above. To quantify immunoreactivity, identical acquisition settings were used for imaging each brain section from all groups within an experiment. The numbers of immunoreactivity-positive soma analysis and intensity of immunoreaction were quantified in 3D stack volumes after uniform background subtraction using the NIS Elements AR software (Nikon) and FIJI (National Institute of Health, USA).

Antibodies for immunostaining were as follows: HRD1 (rabbit, 1:500, homemade), GRP78 BiP (rabbit, 1:500, Abcam; catalog ab21685), α-MSH (sheep, 1:2,000, Millipore; catalog AB5087), β-endorphin (rabbit, 1:2,000, Phoenix Pharmaceuticals; catalog H-022–33, provided by Carol Elisa), and GFP (chicken IgY, 1:300, Abcam; catalog ab13970), p-Y705 STAT3 (rabbit, 1:200, Cell Signaling Technology; catalog 9145), GFAP (rabbit, 1:500, Agilent; Z033429–2), FLAG (mouse, 1:500, Sigma-Aldrich; catalog F-1804), KDEL (rabbit, 1:500, Novus Biologicals; catalog NBP2–75549), eIF3η (goat, 1:500, Santa Cruz Biotechnology; catalog sc-16377). Secondary antibodies for fluorescent immunostaining (all 1:500) were as follows: Anti-rabbit IgG Alexa Fluor 647; anti-goat IgG Alexa Fluor 488 & 647; anti-sheep IgG Cy5 were from Jackson ImmunoResearch. Donkey anti-mouse IgG Alexa flour 555 was from Invitrogen (catalog A32773) and goat anti-chicken IgY FITC was from Aves Labs (catalog F-1005).

### Plasmids.

Mouse LepRb cDNA was provided by Dr. Martin Myer at University of Michigan Medical School. The *LepRb* coding region was amplified by PCR using a primer set containing HindIII and XbaI restriction site at 5’ and 3’ respectively.

F: 5′- CCG AAGCTT ATGATGTGTCAGAAATTCTATGTGGTT-3′

R: 5′- TGC TCTAGA CACAGTTAAGTCACACATCTTATT-3′

Both PCR products and the backbone vector p3xFLAG-CMV14 were digested using HindIII and XbaI restriction enzymes in the double digestion system from New England BioLabs. For construction of LepRb point mutants, quick change mutagenesis was performed using PFU DNA polymerase (600140, Agilent). The following primers were used for mutagenesis to construct LepRb-C602S:

F: 5’- CCTGCTGGTGTCAGACCTCAGTGCAGTCTATG-3’

R: 5’- CATAGACTGCACTGAGGTCTGACACCAGCAGG-3’

### CRISPR/Cas9-based knockout (KO) in HEK293T cells.

HEK293T cells were cultured at 37°C with 5% CO_2_ in DMEM with 10% fetal bovine serum (Fisher Scientific). To generate HRD1deficient HEK293T cells, sgRNA oligonucleotides designed for human *HRD1* (5’-GGACAAAGGCCTGGATGTAC-3’) was inserted into lentiCRISPR v2 (plasmid 52961, Addgene). Cells transfected with empty plasmids without sgRNA were used as wild type control. Cells grown in 10 cm petri dishes were transfected with indicated plasmids using 5μl 1 mg/ml polyethylenimine (PEI, Sigma) per 1μg of plasmids for HEK293T cells. Cells were cultured 24 hours after transfection in medium containing 2 μg/ml puromycin for 48 hours and then in normal growth media.

### Statistics.

Results are expressed as the mean ± SEM unless otherwise stated. Statistical analyses were performed in GraphPad Prism version 8.0 (GraphPad Software Inc.). Comparisons between the groups were made by unpaired two-tailed Student’s t test for two groups, or one-way ANOVA or two-way ANOVA followed by multiple comparisons test for more than two groups. *P* value < 0.05 was considered as statistically significant. All experiments were repeated at least twice and/or performed with several independent biological samples, and representative data are shown.

### Study Approval.

All experiments performed with mice were in compliance with University of Michigan (Ann Arbor, MI) Institutional Animal Care and Use Committee (#PRO00006888) guidelines.

## Data Availability

The materials and reagents used are either commercially available or available upon the request, with detailed information included in Methods. The predicted structure of mLepRb is available at AlphaFold ID AF-P48356-F1. All data supporting the findings and materials for the manuscript are available within the article and the Supplementary Information.

## References

[1] McLean F. H. A high-fat diet induces rapid changes in the mouse hypothalamic proteome. Nutr Metab (Lond) 16, 26, doi:10.1186/s12986-019-0352-9 (2019). 31168311 PMC6489262

[2] Dalvi P. S. High fat induces acute and chronic inflammation in the hypothalamus: effect of high-fat diet, palmitate and TNF-alpha on appetite-regulating NPY neurons. Int J Obes (Lond) 41, 149–158, doi:10.1038/ijo.2016.183 (2017). 27773938

[3] Beutler L. R. Obesity causes selective and long-lasting desensitization of AgRP neurons to dietary fat. Elife 9, doi:10.7554/eLife.55909 (2020). PMC7398661 32720646

[4] Horvath T. L. Synaptic input organization of the melanocortin system predicts diet-induced hypothalamic reactive gliosis and obesity. Proc Natl Acad Sci U S A 107, 14875–14880, doi:10.1073/pnas.1004282107 (2010). 20679202 PMC2930476

[5] Souza G. F. Defective regulation of POMC precedes hypothalamic inflammation in diet-induced obesity. Sci Rep 6, 29290, doi:10.1038/srep29290 (2016). 27373214 PMC4931679

[6] Poon K. Behavioral Feeding Circuit: Dietary Fat-Induced Effects of Inflammatory Mediators in the Hypothalamus. Front Endocrinol (Lausanne) 11, 591559, doi:10.3389/fendo.2020.591559 (2020). 33324346 PMC7726204

[7] Velloso L. A. & Schwartz M. W. Altered hypothalamic function in diet-induced obesity. Int J Obes (Lond) 35, 1455–1465, doi:10.1038/ijo.2011.56 (2011). 21386802 PMC3383790

[8] Zhang K. & Kaufman R. J. From endoplasmic-reticulum stress to the inflammatory response. Nature 454, 455–462, doi:10.1038/nature07203 (2008). 18650916 PMC2727659

[9] Chaudhari N., Talwar P., Parimisetty A., Lefebvre d’Hellencourt C. & Ravanan P. A molecular web: endoplasmic reticulum stress, inflammation, and oxidative stress. Front Cell Neurosci 8, 213, doi:10.3389/fncel.2014.00213 (2014). 25120434 PMC4114208

[10] Zhang Y. Synergistic mechanism between the endoplasmic reticulum and mitochondria and their crosstalk with other organelles. Cell Death Discov 9, 51, doi:10.1038/s41420-023-01353-w (2023). 36759598 PMC9911404

[11] Wu H., Carvalho P. & Voeltz G. K. Here, there, and everywhere: The importance of ER membrane contact sites. Science 361, doi:10.1126/science.aan5835 (2018). PMC6568312 30072511

[12] Kornmann B. An ER-mitochondria tethering complex revealed by a synthetic biology screen. Science 325, 477–481, doi:10.1126/science.1175088 (2009). 19556461 PMC2933203

[13] Rowland A. A. & Voeltz G. K. Endoplasmic reticulum-mitochondria contacts: function of the junction. Nat Rev Mol Cell Biol 13, 607–625, doi:10.1038/nrm3440 (2012). 22992592 PMC5111635

[14] Marchi S., Patergnani S. & Pinton P. The endoplasmic reticulum-mitochondria connection: one touch, multiple functions. Biochim Biophys Acta 1837, 461–469, doi:10.1016/j.bbabio.2013.10.015 (2014). 24211533

[15] Ozcan L. Endoplasmic reticulum stress plays a central role in development of leptin resistance. Cell Metab 9, 35–51, doi:10.1016/j.cmet.2008.12.004 (2009). 19117545

[16] Purkayastha S. Neural dysregulation of peripheral insulin action and blood pressure by brain endoplasmic reticulum stress. Proc Natl Acad Sci U S A 108, 2939–2944, doi:10.1073/pnas.1006875108 (2011). 21282643 PMC3041145

[17] Ramirez S. & Claret M. Hypothalamic ER stress: A bridge between leptin resistance and obesity. FEBS Lett 589, 1678–1687, doi:10.1016/j.febslet.2015.04.025 (2015). 25913783

[18] Schneeberger M. Mitofusin 2 in POMC neurons connects ER stress with leptin resistance and energy imbalance. Cell 155, 172–187, doi:10.1016/j.cell.2013.09.003 (2013). 24074867 PMC3839088

[19] Ye Z., Liu G., Guo J. & Su Z. Hypothalamic endoplasmic reticulum stress as a key mediator of obesity-induced leptin resistance. Obes Rev 19, 770–785, doi:10.1111/obr.12673 (2018). 29514392

[20] Zhang X. Hypothalamic IKKbeta/NF-kappaB and ER stress link overnutrition to energy imbalance and obesity. Cell 135, 61–73, doi:10.1016/j.cell.2008.07.043 (2008). 18854155 PMC2586330

[21] Yao T. Ire1alpha in Pomc Neurons Is Required for Thermogenesis and Glycemia. Diabetes 66, 663–673, doi:10.2337/db16-0533 (2017). 28028078 PMC5319716

[22] Xiao Y. Knockout of inositol-requiring enzyme 1alpha in pro-opiomelanocortin neurons decreases fat mass via increasing energy expenditure. Open Biol 6, doi:10.1098/rsob.160131 (2016). PMC5008012 27558934

[23] Williams K. W. Xbp1s in Pomc neurons connects ER stress with energy balance and glucose homeostasis. Cell Metab 20, 471–482, doi:10.1016/j.cmet.2014.06.002 (2014). 25017942 PMC4186248

[24] Friedlander R., Jarosch E., Urban J., Volkwein C. & Sommer T. A regulatory link between ER-associated protein degradation and the unfolded-protein response. Nat Cell Biol 2, 379–384, doi:10.1038/35017001 (2000). 10878801

[25] Qi L., Tsai B. & Arvan P. New Insights into the Physiological Role of Endoplasmic Reticulum-Associated Degradation. Trends Cell Biol 27, 430–440, doi:10.1016/j.tcb.2016.12.002 (2017). 28131647 PMC5440201

[26] Travers K. J. Functional and genomic analyses reveal an essential coordination between the unfolded protein response and ER-associated degradation. Cell 101, 249–258, doi:10.1016/s0092-8674(00)80835-1 (2000). 10847680

[27] Hwang J. & Qi L. Quality Control in the Endoplasmic Reticulum: Crosstalk between ERAD and UPR pathways. Trends Biochem Sci 43, 593–605, doi:10.1016/j.tibs.2018.06.005 (2018). 30056836 PMC6327314

[28] Carvalho P., Goder V. & Rapoport T. A. Distinct ubiquitin-ligase complexes define convergent pathways for the degradation of ER proteins. Cell 126, 361–373, doi:10.1016/j.cell.2006.05.043 (2006). 16873066

[29] Gardner R. G. Endoplasmic reticulum degradation requires lumen to cytosol signaling. Transmembrane control of Hrd1p by Hrd3p. J Cell Biol 151, 69–82 (2000). 11018054 10.1083/jcb.151.1.69 PMC2189800

[30] Hampton R. Y., Gardner R. G. & Rine J. Role of 26S proteasome and HRD genes in the degradation of 3-hydroxy-3-methylglutaryl-CoA reductase, an integral endoplasmic reticulum membrane protein. Mol Biol Cell 7, 2029–2044, doi:10.1091/mbc.7.12.2029 (1996). 8970163 PMC276048

[31] Bhattacharya A. & Qi L. ER-associated degradation in health and disease - from substrate to organism. J Cell Sci 132, doi:10.1242/jcs.232850 (2019). PMC6918741 31792042

[32] Vashistha N., Neal S. E., Singh A., Carroll S. M. & Hampton R. Y. Direct and essential function for Hrd3 in ER-associated degradation. Proc Natl Acad Sci U S A 113, 5934–5939, doi:10.1073/pnas.1603079113 (2016). 27170191 PMC4889393

[33] Wu X. & Rapoport T. A. Mechanistic insights into ER-associated protein degradation. Curr Opin Cell Biol 53, 22–28, doi:10.1016/j.ceb.2018.04.004 (2018). 29719269 PMC6131047

[34] Schoebel S. Cryo-EM structure of the protein-conducting ERAD channel Hrd1 in complex with Hrd3. Nature 548, 352–355, doi:10.1038/nature23314 (2017). 28682307 PMC5736104

[35] Sha H. The ER-associated degradation adaptor protein Sel1L regulates LPL secretion and lipid metabolism. Cell Metab 20, 458–470, doi:10.1016/j.cmet.2014.06.015 (2014). 25066055 PMC4156539

[36] Wu S. A. The mechanisms to dispose of misfolded proteins in the endoplasmic reticulum of adipocytes. Nat Commun 14, 3132, doi:10.1038/s41467-023-38690-4 (2023). 37253728 PMC10229581

[37] Bhattacharya A. Hepatic Sel1L-Hrd1 ER-associated degradation (ERAD) manages FGF21 levels and systemic metabolism via CREBH. EMBO J 37, doi:10.15252/embj.201899277 (2018). PMC6236331 30389665

[38] Wei J. HRD1-ERAD controls production of the hepatokine FGF21 through CREBH polyubiquitination. EMBO J 37, doi:10.15252/embj.201898942 (2018). PMC6236336 30389664

[39] Kim G. H. Hypothalamic ER-associated degradation regulates POMC maturation, feeding, and age-associated obesity. J Clin Invest 128, 1125–1140, doi:10.1172/JCI96420 (2018). 29457782 PMC5824855

[40] Shi G. ER-associated degradation is required for vasopressin prohormone processing and systemic water homeostasis. J Clin Invest 127, 3897–3912, doi:10.1172/JCI94771 (2017). 28920920 PMC5617659

[41] Yoshida S. Endoplasmic reticulum-associated degradation is required for nephrin maturation and kidney glomerular filtration function. J Clin Invest 131, doi:10.1172/JCI143988 (2021). PMC8011890 33591954

[42] Shrestha N. Sel1L-Hrd1 ER-associated degradation maintains beta cell identity via TGF-beta signaling. J Clin Invest 130, 3499–3510, doi:10.1172/JCI134874 (2020). 32182217 PMC7324191

[43] Toda C., Santoro A., Kim J. D. & Diano S. POMC Neurons: From Birth to Death. Annu Rev Physiol 79, 209–236, doi:10.1146/annurev-physiol-022516-034110 (2017). 28192062 PMC5669621

[44] Bumaschny V. F. Obesity-programmed mice are rescued by early genetic intervention. J Clin Invest 122, 4203–4212, doi:10.1172/JCI62543 (2012). 23093774 PMC3484438

[45] Munzberg H., Huo L., Nillni E. A., Hollenberg A. N. & Bjorbaek C. Role of signal transducer and activator of transcription 3 in regulation of hypothalamic proopiomelanocortin gene expression by leptin. Endocrinology 144, 2121–2131, doi:10.1210/en.2002-221037 (2003). 12697721

[46] Liu H., Du T., Li C. & Yang G. STAT3 phosphorylation in central leptin resistance. Nutr Metab (Lond) 18, 39, doi:10.1186/s12986-021-00569-w (2021). 33849593 PMC8045279

[47] Baumann H. The full-length leptin receptor has signaling capabilities of interleukin 6-type cytokine receptors. Proc Natl Acad Sci U S A 93, 8374–8378, doi:10.1073/pnas.93.16.8374 (1996). 8710878 PMC38678

[48] Chen H. Evidence that the diabetes gene encodes the leptin receptor: identification of a mutation in the leptin receptor gene in db/db mice. Cell 84, 491–495, doi:10.1016/s0092-8674(00)81294-5 (1996). 8608603

[49] Uotani S., Bjorbaek C., Tornoe J. & Flier J. S. Functional properties of leptin receptor isoforms: internalization and degradation of leptin and ligand-induced receptor downregulation. Diabetes 48, 279–286, doi:10.2337/diabetes.48.2.279 (1999). 10334302

[50] Plemper R. K. Genetic interactions of Hrd3p and Der3p/Hrd1p with Sec61p suggest a retro-translocation complex mediating protein transport for ER degradation. J Cell Sci 112 (Pt 22), 4123–4134, doi:10.1242/jcs.112.22.4123 (1999). 10547371

[51] Sun S. Sel1L is indispensable for mammalian endoplasmic reticulum-associated degradation, endoplasmic reticulum homeostasis, and survival. Proc Natl Acad Sci U S A 111, E582–591, doi:10.1073/pnas.1318114111 (2014). 24453213 PMC3918815

[52] Cao L. Global site-specific analysis of glycoprotein N-glycan processing. Nat Protoc 13, 1196–1212, doi:10.1038/nprot.2018.024 (2018). 29725121 PMC5941933

[53] Nunziata A. Functional and Phenotypic Characteristics of Human Leptin Receptor Mutations. J Endocr Soc 3, 27–41, doi:10.1210/js.2018-00123 (2019). 30560226 PMC6293235

[54] Saeed S. Genetic variants in LEP, LEPR, and MC4R explain 30% of severe obesity in children from a consanguineous population. Obesity (Silver Spring) 23, 1687–1695, doi:10.1002/oby.21142 (2015). 26179253

[55] Saeed S. High morbidity and mortality in children with untreated congenital deficiency of leptin or its receptor. Cell Rep Med 4, 101187, doi:10.1016/j.xcrm.2023.101187 (2023). 37659411 PMC10518629

[56] Peelman F., Zabeau L., Moharana K., Savvides S. N. & Tavernier J. 20 years of leptin: insights into signaling assemblies of the leptin receptor. J Endocrinol 223, T9–23, doi:10.1530/JOE-14-0264 (2014). 25063754

[57] Moharana K. Structural and mechanistic paradigm of leptin receptor activation revealed by complexes with wild-type and antagonist leptins. Structure 22, 866–877, doi:10.1016/j.str.2014.04.012 (2014). 24882746

[58] Tsirigotaki A. Mechanism of receptor assembly via the pleiotropic adipokine Leptin. Nat Struct Mol Biol 30, 551–563, doi:10.1038/s41594-023-00941-9 (2023). 36959263

[59] Zhou Z. Endoplasmic reticulum-associated degradation regulates mitochondrial dynamics in brown adipocytes. Science 368, 54–60, doi:10.1126/science.aay2494 (2020). 32193362 PMC7409365

[60] Hosoi T. Endoplasmic reticulum stress induces leptin resistance. Mol Pharmacol 74, 1610–1619, doi:10.1124/mol.108.050070 (2008). 18755873

[61] Heifetz A., Keenan R. W. & Elbein A. D. Mechanism of action of tunicamycin on the UDP-GlcNAc:dolichyl-phosphate Glc-NAc-1-phosphate transferase. Biochemistry 18, 2186–2192, doi:10.1021/bi00578a008 (1979). 444447

[62] Qi L., Yang L. & Chen H. Detecting and quantitating physiological endoplasmic reticulum stress. Methods Enzymol 490, 137–146, doi:10.1016/B978-0-12-385114-7.00008-8 (2011). 21266248 PMC3374842

[63] Yang L. A Phos-tag-based approach reveals the extent of physiological endoplasmic reticulum stress. PLoS One 5, e11621, doi:10.1371/journal.pone.0011621 (2010). 20661282 PMC2905412

